# Do Developmental Constraints and High Integration Limit the Evolution of the Marsupial Oral Apparatus?

**DOI:** 10.1093/icb/icw039

**Published:** 2016-06-02

**Authors:** Anjali Goswami, Marcela Randau, P. David Polly, Vera Weisbecker, C. Verity Bennett, Lionel Hautier, Marcelo R. Sánchez-Villagra

**Affiliations:** *Department of Genetics, Evolution and Environment, University College London, Gower Street, London, WC1E 6BT, UK; ^†^Department of Earth Sciences, University College London, Gower Street, London, WC1E 6BT, UK; ^‡^Department of Geological Sciences, 1001 E. 10th Street, Indiana University, Bloomington, IN, 47405, USA; ^§^School of Biological Sciences, Goddard Building 8, University of Queensland, St. Lucia 4072, Australia; ^¶^Laboratoire de Paléontologie, Institut des Sciences de l'Èvolution de Montpellier (CNRS, UM, IRD, EPHE), c.c. 064, Université Montpellier 2, Place Eugène Bataillon, F-34095 Montpellier, Cedex 5 , France; ^‖^Palaeontological Institute and Museum, University of Zürich, Karl-Schmid-Strasse 4, CH-8006, Zürich, Switzerland

## Abstract

Developmental constraints can have significant influence on the magnitude and direction of evolutionary change, and many studies have demonstrated that these effects are manifested on macroevolutionary scales. Phenotypic integration, or the strong interactions among traits, has been similarly invoked as a major influence on morphological variation, and many studies have demonstrated that trait integration changes through ontogeny, in many cases decreasing with age. Here, we unify these perspectives in a case study of the ontogeny of the mammalian cranium, focusing on a comparison between marsupials and placentals. Marsupials are born at an extremely altricial state, requiring, in most cases, the use of the forelimbs to climb to the pouch, and, in all cases, an extended period of continuous suckling, during which most of their development occurs. Previous work has shown that marsupials are less disparate in adult cranial form than are placentals, particularly in the oral apparatus, and in forelimb ontogeny and adult morphology, presumably due to functional selection pressures on these two systems during early postnatal development. Using phenotypic trajectory analysis to quantify prenatal and early postnatal cranial ontogeny in 10 species of therian mammals, we demonstrate that this pattern of limited variation is also apparent in the development of the oral apparatus of marsupials, relative to placentals, but not in the skull more generally. Combined with the observation that marsupials show extremely high integration of the oral apparatus in early postnatal ontogeny, while other cranial regions show similar levels of integration to that observed in placentals, we suggest that high integration may compound the effects of the functional constraints for continuous suckling to ultimately limit the ontogenetic and adult disparity of the marsupial oral apparatus throughout their evolutionary history.

## Introduction

Why some clades achieve immense taxonomic, morphological, or ecological diversity while other, often closely related, clades are modest or poor in some or all of these measures is a question that has interested evolutionary biologists for centuries. Attempts to understand this phenomenon can focus either on the successful clade, perhaps identifying a key innovation or new opportunity that allowed for its radiation, or on the depauperate one, testing for evidence of a developmental constraint or lack of ecological opportunity that has limited its ability to evolve as quickly or as much. Key innovations are usually viewed as promoting the diversification of a clade, but they may just as frequently, or even more frequently, limit its phenotypic evolution because of functional specialization (Asher and Sánchez-Villagra 2005; Wainwright 2016, this volume).

### Ontogeny, disparity, and the marsupial–placental dichotomy

An example of a group that is limited by such an innovation may be the mammalian clade Marsupialia. This group of ∼350 extant species is the sister clade to our own clade, Placentalia, which numbers over 5000 species (Wilson and Reeder 2005). The two clades are distinguished by their divergent reproductive strategies. Placentals give birth to relatively well-developed young, with most nervous and somatic systems in place by birth and postnatal development mainly involving growth in size. Marsupials, in contrast, give birth after a very short period of gestation to extremely altricial young, which have rudimentary nervous systems and only the musculoskeletal systems of the oral apparatus and the anterior postcranium, particularly the forelimbs, well developed at birth (Lillegraven et al. 1987; Tyndale-Biscoe and Renfree 1987; Smith 1997, 2001, 2002, 2006; Nunn and Smith 1998; Maier 1999; Gemmell et al. 2000; Sears 2004). Most of their development occurs instead during a lengthy postnatal period of lactation; in particular, they reside at their mother’s teat and suckle continuously during early postnatal development. Although the marsupial strategy has traditionally been viewed as intermediate between that of the egg-laying mammals, monotremes, and that of placentals (Young 1957), more recent analyses instead suggest that it is the marsupial system that is more derived (Weisbecker et al. 2008; Smith 2013, 2015). Concerning the postcranial skeleton, for example, there are more autapomorphic shifts in skeletal ossification detected among marsupials than placentals (Weisbecker et al. 2008).

These differences in development between marsupials and placentals have long been linked to their disparate evolutionary histories (Lillegraven 1975; Lillegraven et al. 1987). The necessity for marsupial neonates, who are born between two weeks and a month after conception, to crawl to their mother’s pouch has been hypothesized to limit the evolutionary lability of the forelimb, which must be functional as a climbing limb at this early stage of development. This requirement manifests itself in the high level of ossification of the marsupial forelimb at birth, relative to the hind limb (Sánchez-Villagra 2002; Weisbecker et al. 2008), and in the limited variation in ontogenetic trajectories of forelimb shape change in marsupials, relative to placentals (Sears 2004). Furthermore, marsupial forelimbs display reduced adult disparity and evolutionary rates relative to marsupial hind limbs, which ossify after birth, and relative to placental forelimbs (Sears 2004; Cooper and Steppan 2010; Kelly and Sears 2011a).

**Fig. 1:**
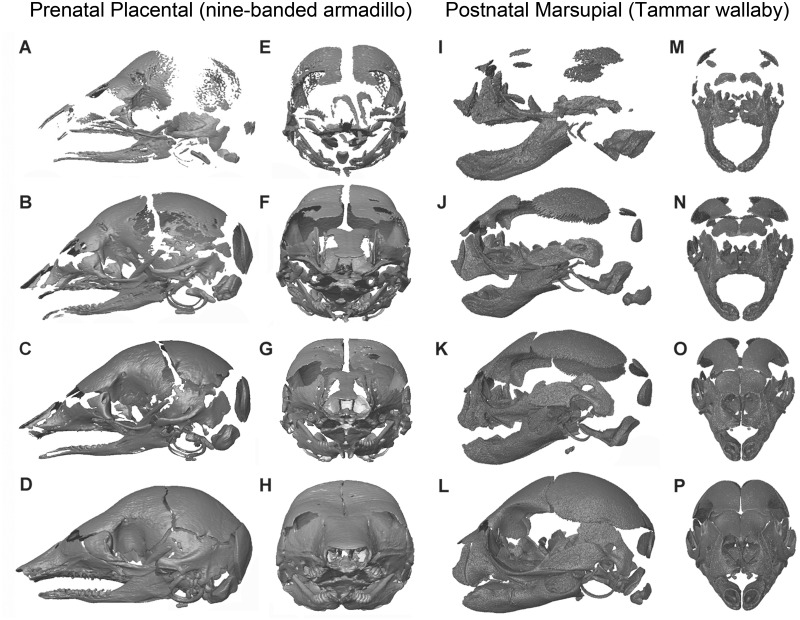
Comparative skull ossification for four prenatal stages of placental, *Dasypus novemcinctus*, the nine-banded armadillo (A–H), and four postnatal stages of a marsupial, *Macropus eugenii*, the Tammar wallaby (I–P), demonstrating the clear differences level of cranial ossification of birth. All of the prenatal armadillo stages (A–H) show greater ossification of the skull than the earliest postnatal stage of the wallaby (I, M), and the latest sampled prenatal armadillo (D, H) is more ossified than a 6–8 week old wallaby (L, P; wallaby age estimate from Ramirez-Chaves et al. 2016). (A)–(D) and (I)–(L) are lateral views, and (E)–(H) and (M)–(P) are anterior views, proceeding from earliest to latest stages sampled from top to bottom. *Dasypus novemcinctus* specimens are: (A) and (E), 85893b; (B) and (F), 12XII01a; (C) and (G), A5022; (D) and (H), 40647. *Macropus eugenii* specimens are: (I) and (M), Meug1621; (J) and (N), Meug1694; (K) and (O), Meug1682_Yellow; and (L) and (P), Meug1716_yellow.

There are similar differences in timing of ossification in marsupial cranial elements, with the bones of the oral apparatus forming first, in advance of birth (Fig. 1). These bones are involved in the continuous suckling that begins immediately after birth and extends for several months (Tyndale-Biscoe and Renfree 1987; German and Crompton 1996; Gemmell et al. 2000), imposing a far greater functional pressure on these elements than that experienced by any placental infant. Most other cranial bones do not begin to ossify until after birth (German and Crompton 1996; Smith 1997, 2006). This shift in timing and level of development has also been linked to reduced disparity of the adult marsupial cranium, relative to that of placentals. More specifically, the early developing bones of the oral apparatus show significantly less disparity than those of placentals, but the later developing regions of the skull (i.e., the neurocranium) importantly show no difference in disparity compared to placentals (Bennett and Goswami 2013).

### Phenotypic integration, mammalian development, and morphological evolution

In addition to the potential developmental constraints imposed by functional pressures during early ontogeny discussed above, the observed differences in marsupial and placental skeletal development have also previously been tied to patterns of phenotypic integration in the cranium and postcranium (Maunz and German 1996; Goswami et al. 2009, 2012; Bennett and Goswami 2011; Kelly and Sears 2011b). The relationships among phenotypic traits have long been hypothesized to reflect genetic, developmental, and functional interactions among traits. These relationships, termed phenotypic integration, can be identified through quantitative analysis of traits, which has often indicated that anatomical structures are modular, meaning that they form subsets of highly correlated traits that are relatively independent of other traits or sets of traits. Integration and modularity can be analyzed at multiple scales, the most common being variational integration (Armbruster et al. 2014; Klingenberg forthcoming 2014), which focuses on the species- or population-level and samples a single ontogenetic stage (typically adults). Where possible, one can also investigate how integration changes through ontogeny, usually by sampling many specimens from individual ontogenetic stages (Zelditch 1988; Zelditch and Carmichael 1989a, 1989b; Willmore et al. 2006; Goswami and Polly 2010b; Goswami et al. 2012).

**Fig. 2:**
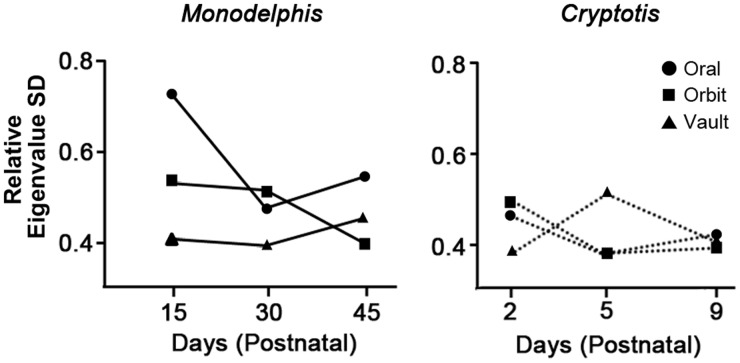
Magnitude of integration through ontogeny in *Monodelphis* (left) and *Cryptotis* (right) cranial regions, as measured by relative eigenvalue standard deviation of the congruence coefficient, demonstrating the high integration of the oral region in the early postnatal ontogeny of the sampled marsupial, *Monodelphis domestica* (modified from Goswami et al. 2012).

Previous studies of both variational and ontogenetic integration and modularity across therian mammals have suggested a link between developmental strategy and patterns of integration. In the single study of cranial integration through ontogeny comparing marsupials and placentals, Goswami et al. (2012) demonstrated that the marsupial *Monodelphis domestica* displayed much higher integration among oral bones in the earliest postnatal stage sampled (15 days postnatal) than was observed in any other cranial region for that taxon or any cranial region of the sampled placental, *Cryptotis parva* (Fig. 2). As large sample sizes for well-staged non-model organisms are difficult to obtain, it is not possible to ascertain if this pattern applies to other marsupials, but we suggested that this high integration of the oral apparatus early in postnatal ontogeny may reflect the need for strong coordination of these elements during a period when marsupial young are suckling constantly or near-constantly.

In the postcranium, the heterochronic differences in limb bone ossification discussed above are reflected in different patterns of variational modularity in long bones of adult marsupials, placentals, and monotremes (Bennett and Goswami 2011; Kelly and Sears 2011b; Weisbecker 2011). Marsupials and placentals also display different patterns of modularity in postcranial ossification timing, as demonstrated by rank correlation analysis of developmental sequences (Goswami et al. 2009), which identifies coordination of heterochronic shifts among elements (Poe 2004). This concordance of developmental strategy and postcranial modularity opens up the possibility of identifying when these developmental strategies evolved in therian mammal evolution, via studies of fossil specimens, potentially allowing for more accurate reconstructions of the selection pressures that drove their evolution (Bennett and Goswami 2011; Goswami et al. 2015).

Patterns of integration and modularity are important to characterize and understand for multiple reasons; perhaps most fundamentally, trait integration is hypothesized to be a major influence on the evolution of individual traits (Wagner and Altenberg 1996; Klingenberg 2004). Simulations have shown that pattern and magnitude of trait integration can influence the magnitude and direction of response to selection, by redirecting variance in preferred directions and away from the direction of selection (Hansen and Houle 2008; Marroig et al. 2009; Shirai and Marroig 2010; Goswami et al. 2014). On a macroevolutionary scale, this effect can be expected to limit evolution in directions away from the principal axes of variation for a given sample, but also increase the range of morphological disparity or evolutionary rates along those preferred axes (Goswami et al. 2014). Ultimately, trait integration can produce both less and more disparity, or divergence, than expected under models of no integration, depending on the relationship between selection pressures and principal axes of variation (fig. 6 in Goswami et al. 2014).

Expanding the results of these simulations to empirical datasets is difficult, because many other factors can limit clade disparity or evolutionary rates, or our estimation of these variables, including ecological opportunity and past extinction events. Nonetheless, there have been a few attempts to analyze the relationship between trait integration and disparity or rates in diverse clades. A study of mammalian crania compared trait variances of highly integrated and weakly integrated regions of the skull and found some support that high integration was associated with lower disparity (Goswami and Polly 2010a). Unexpectedly, analysis of that same dataset suggested that there was no relationship between magnitude of integration and evolutionary rates, with some of the most highly integrated regions (e.g., the basicranium) showing low disparity across taxa, but some of the highest rates of evolution (Goswami et al. 2014). Another study of cranial shape across mammals showed a similar result that evolutionary rates and evolvability were not positively correlated (Linde-Medina et al. forthcoming 2016), while a simulation study using empirically-derived marsupial cranial covariance structures suggested that their evolution may be influenced mainly by size variation due to high magnitudes of integration (Shirai and Marroig 2010). In contrast, studies of non-mammalian clades have found higher rates of evolution and higher disparity in taxa with greater modularity, suggesting that lower overall integration may be associated with faster evolution (Claverie and Patek 2013; Collar et al. 2014). A large-scale study of crinoids found no correlation between level of integration and clade disparity, but rather that shifts in the pattern of integration were associated with changes in disparity (Gerber 2013). Clearly, much more work is needed to understand the macroevolutionary consequences of phenotypic integration, but most studies have suggested that trait integration and modularity can significantly impact morphological evolution.

### Aims and objectives

Here, we conduct the first large-scale comparative analysis of cranial ontogenetic trajectories across extant marsupials and placentals to determine whether patterns of skull development support the hypothesis that the limited diversity of the masticatory apparatus observed in adult marsupials, relative to placentals, is rooted in constrained development of these structures. We specifically hypothesize that the similarities in ontogenetic trajectories observed for the marsupial forelimb will be replicated in the early ossifying premaxilla, maxilla, and dentary, which constitute that part of the head skeleton presumably under strong functional pressures for continuous suckling immediately after birth.

## Methods

### Specimens

Ontogenetic series for non-model organisms are often difficult to obtain for the period spanning late prenatal and early postnatal development, so very few studies have examined both (Wilson 2011). We gathered four or more stages spanning early postnatal ontogeny for 10 species (Table 1), including six placentals (*Cavia porcellus, Peromyscus melanophrys, Talpa europaea, Rousettus amplexicaudatus, Llama guanicoe*, and *Dasypus novemcinctus*) and four marsupials (*M. domestica, Macropus eugenii, Trichosurus vulpecula*, and *Phascolarctos cinereus*). Placental specimens represent three of the four superorders, Xenarthra, Laurasiatheria, and Euarchontoglires, while marsupial specimens represent the two most diverse superorders, Diprotodontia and Didelphimorphia (Wilson and Reeder 2005; Bininda-Emonds et al. 2007). Sources of specimens range from lab-reared animals (e.g., *Monodelphis*) to colony-reared specimens from planned culls (e.g., *Macropus*) to specimens harvested for traditional ceremonies (*Llama*) to historical collections (e.g., *Rousettus*). Exact age information was only available for lab-reared specimens (*Monodelphis, Cavia*). All specimens, however, span an overlapping period of craniogenesis, ranging from when most cranial bones have begun to ossify to the early formation of suture boundaries (Fig. 1). This period is entirely postnatal for all marsupial specimens, but encompasses prenatal and early postnatal stages in placentals. Specimens without age information were placed in rank order based either on crown-rump length (*Dasypus*) or skull length. The total dataset includes 76 specimens, with the smallest samples (4 stages) for *Trichosurus* and *Cavia*, and the largest (13 stages) for *Macropus*. All specimens are detailed in Supplementary Table S1.

**Table 1:** Species, specimens, and trajectory sizes from phenotypic trajectory analysis of all cranial measurements (with PC1), oral measurements only (with PC1), and oral measurements only (without PC1)

	Specimens	All cranial measurements	Oral apparatus	Oral apparatus with out PCO1
Marsupials				
*Macropus eugenii*	13	2.976	0.448	0.437
*Trichosurus vulpecula*	4	4.014	0.611	0.609
*Phascolarctos cinereus*	9	3.939	0.939	0.935
*Monodelphis domestica*	8	3.437	1.120	1.079
Placentals				
*Dasypus novemcinctus*	8	3.894	1.634	1.618
*Peromyscus melanophrys*	9	3.602	1.547	1.537
*Cavia porcellus*	5	3.702	1.787	1.773
*Talpa europaea*	9	3.679	1.801	1.790
*Roussetus amplexicaudatus*	5	3.937	2.248	2.206
*Llama guanicoe*	6	2.955	1.117	1.088

*Note:* As ranks were not significantly different for the all cranial measurements analysis without PC1, those trajectories are not reported here.

### Data collection

Three-dimensional landmarks were collected from specimens using three approaches, depending on the condition of specimens and availability of scanners. *Cavia, Dasypus, Macropus, Phascolarctos*, and *Trichosurus* were micro-CT scanned at the University of Cambridge, University College London, the Royal Veterinary College (London), Helmholtz-Zentrum (Berlin), and the Natural History Museum (London) and then digitized using Avizo 7.0 (FEI, Hillsboro, Oregon). Cleared-and-stained specimens of *Rousettus, Peromyscus, Talpa*, and *Monodelphis* were digitized using Reflex Measurement microscopes (Consultantnet Ltd, Fowlmere, UK) housed at the University of Zurich, Queen Mary College, and Indiana University. *Llama* specimens were digitized with an Immersion Microscribe 3-D digitizer (Immersion Corp., San Jose, CA). Because of the different specimen preparations and landmarking tools, landmarks were decomposed into 30 length measurements of individual bones, as well as skull length as a measure of body size (Table 2; Supplementary Table S1). Skull length was used instead of whole body measures, e.g. crown-rump length, because many specimens were obtained as isolated heads or skull scans, and thus lacked the whole body measures. Skull length is also a common measure for body size, particularly in cranial studies (e.g., Flores et al. 2013). As CT reconstructions obtained from different sources were not uniformly scaled in a few cases, all measurements were first divided against skull length for the respective specimen to provide comparable measurements. In some cases, all measurements were not available for every specimen, either because bones had not yet begun to ossify in the earliest stages, were not visible from available views (limited to some specimens gathered with Reflex microscopy), or had already fused with adjacent bones in older specimens, making sutures impossible to identify. In total, 119 out of 2280 measurements, or 5.22%, were missing from the final dataset.

**Table 2:** List and descriptions of measurements used in analyses

Measurements	Description
Nasal midline length	From anteromedial extreme to posteromedial extreme in dorsal view
Nasal anterior width	From left to right anterolateral extremes in dorsal view
Nasal posterior width	From left to right posterolateral extremes in dorsal view
*Premaxilla lateral length	From anteromedial extreme to posteroventral extreme in lateral view
*Premaxilla posterior height	From posteroventral extreme to posterodorsal extreme in lateral view
*Maxilla anterior height	From anteroventral extreme to anterodorsal extreme in lateral view
*Maxilla posterior height	From the posteroventral extreme (usually ventral suture with jugal, where present) to posterodorsal extreme in lateral view
*Maxilla lateral length	From anteroventral extreme to posteroventral extreme (usually ventral suture with jugal, where present) in lateral view
Jugal ventral length	From anteroventral extreme (usually ventral suture with maxilla) to posteroventral tip in ventral view
Squamosal length	From anterodorsal extreme (on zygomatic arch) to posteroventral extreme
Squamosal posterior height	From posteroventral extreme to posterodorsal extreme
Frontal midline length	From anteromedial extreme to posteromedial extreme
Frontal length lateral	From anterolateral extreme to posterolateral extreme
Parietal midline length	From anteromedial extreme to posteromedial extreme
Parietal lateral length	From anterolateral extreme to posterolateral extreme
Supraoccipital midline height	From dorsomedial extreme to the opisthion
Supraoccipital dorsal width	From left to right dorsolateral extremes
Exoccipital ventral width	From posteromedial extreme to posterolateral extreme along ventral edge
Exoccipital dorsal width	From posteromedial extreme to posterolateral extreme along ventral edge
Exoccipital lateral height	From ventrolateral extreme to dorsolateral extreme in posterior view
Palatine midline length	From anteromedial extreme to posteromedial extreme
Palatine posterior width	From left to right posterolateral extremes
Pterygoid length	From anteroventral extreme to posteroventral tip
Basioccipital anterior width	From left to right anterolateral extremes
Basioccipital posterior width	From left to right posterolateral extremes
Basioccipital length	From left anterolateral extreme to left posterolateral extreme
*Dentary body length	From anterodorsal extreme of the body to the dorsal intersection of the body and ramus
*Dentary ramus height	From posterior extreme of the angular process (or posteroventral extreme, if not present) to posterodorsal extreme of the coronoid process
*Dentary ramus length	From posterior extreme of the angular process (or posteroventral extreme, if not present) to ventral intersection of the body and ramus
Skull length	From anteromedial extreme of the premaxilla to the basion

*Note:* *indicates those included in analyses limited to the oral region. Because many sutures are not formed in the early ontogeny, landmarks generally refer to extremal points of bones rather than sutures.

### Data analysis

Cranial measurements were logged prior to all further analysis. Due to missing data, measurements were subjected to Principal Coordinates Analysis (PCO) in PAST 3.0 (Hammer et al. 2001), using the full dataset of 76 specimens, to qualitatively assess overall variation in ontogenetic trajectories. To further quantify differences in ontogenetic trajectories among taxa, phenotypic trajectory analysis, or PTA, (Adams and Collyer 2009) was applied. Because this method requires equal numbers of stages for each group, all species were reduced to the minimum sample size of four ranks. For taxa with more specimens, the subsample was selected to represent the range of ages (or sizes, when age data were not available) of the full sample, while also reducing missing data. A new PCO for the subsampled dataset of 40 specimens was performed in PAST 3.0, and PCO scores were imported into R (R Core Team 2014) for analysis using the “geomorph” package (Adams and Otarola-Castillo 2013). PTA groupings used included data collection type (micro-CT, Reflex microscopy, or 3-D digitizer), to assess possible effects of data collection approach, species, and infraclass (Placentalia and Marsupialia), as well as the default grouping of rank. Trajectory size (the total length of the ontogenetic trajectory across the four sampled stages), direction, and shape were compared for each set of groups, with significance of differences assessed using 1000 residual randomization permutations (Adams and Collyer 2009). Because PCO1 appeared to reflect size in some PCO analyses, and to facilitate comparisons to previous studies of constraints on the marsupial forelimb which also excluded PC1 (Sears 2004), PTA analyses were run with and without PCO1 scores.

To assess the effects of early ossification and functional pressures of suckling on the oral apparatus of marsupials, additional PTA analyses were conducted that were limited to the eight measurements of the premaxilla, maxilla, and dentary. As with the full cranial dataset, analyses were run both with and without PCO1 scores.

## Results

### Principal Coordinates Analysis

PCO analysis of the full dataset demonstrated substantial amounts of variance explained by the first five axes (20.8%, 18.8%, 8.9%, 8.2%, and 6.1%, respectively). The first 15 axes each represented more than 1% of the total variance. PCO1 was defined at the negative end by *M. eugenii*, while *Talpa* and *Peromyscus* occupied the positive end. *Llama* specimens demarcated the negative end of PCO2, with *Talpa* and *Macropus* at its positive end. There was large overlap of marsupials and placentals on all of the first five axes.

**Fig. 3:**
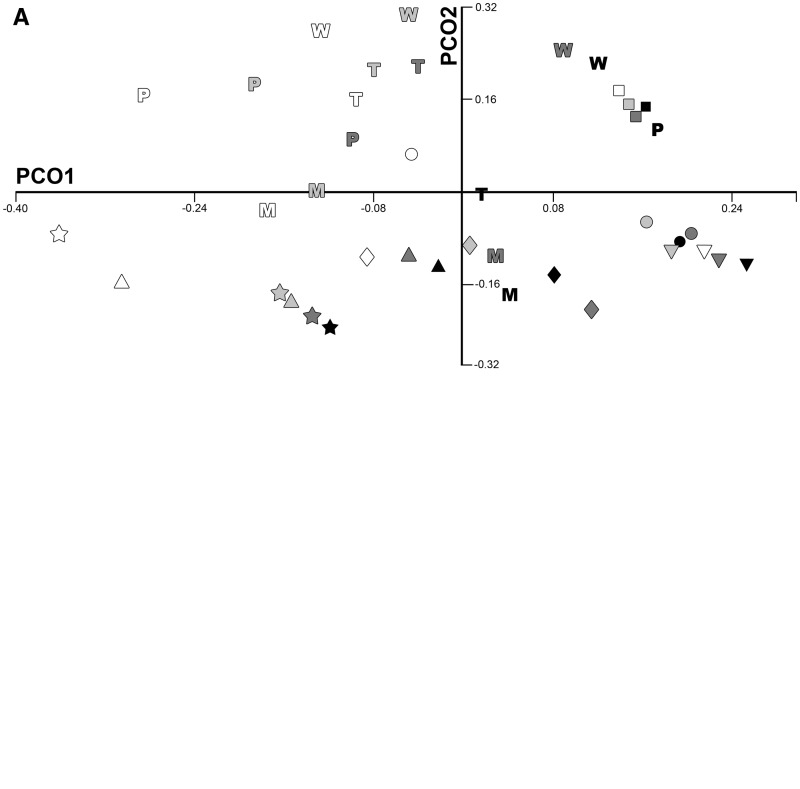
PCO plot of set of four ranks using (A) the full dataset; and (B) the dataset limited to the premaxilla, maxilla, and dentary measurements. Symbols are placentals: star, *Talpa*; triangle; *Rousettus*; diamond, *Peromyscus*; circle, *Dasypus*; square, *Cavia*; inverted triangle, *Llama.* Letters are marsupials: P, *Phascolarctos*; W, *Macropus*; T, *Trichosurus*; M, *Monodelphis*. Shading represents ontogenetic rank, with increasing darkness indicating increasing age (i.e., white denoting the youngest rank and black denoting the oldest rank). In the all cranial measurement dataset (A), both marsupials and placentals are widely distributed and show large shifts in shape through ontogeny, as can be qualitatively assessed by the range of morphospace covered from the youngest (white) to oldest (black) ranks for each species. In contrast, marsupials are limited to a small area of morphospace and show significantly smaller shifts in shape through ontogeny in the analysis of only the early ossifying bones of the oral apparatus (B).

PCO analysis of the subsampled dataset, with four ranks for each species, resulted in relatively high eigenvalues for several axes. PCOs1–6 represented 23.2%, 17.8%, 11.3%, 9.9%, 5.3%, and 4.8% of the total variance, respectively, with the first 15 axes each representing more than 1% of the total variance. PCO1 appeared to be dominated by overall size, with smallest or youngest specimens falling at the negative end of the PCO1, while the largest and oldest specimens were directed toward the positive end of that axis (Fig. 3A). Marsupials and placentals overlapped entirely on the first 15 PCO axes.

When PCO analysis is limited to the premaxilla, maxilla, and dentary, variance was more concentrated in the first axis (42.1%), although PCO axes 2–4 also explained substantial amounts of variance (16.1%, 9.1%, and 7.7%, respectively). All eight axes represented more than 1% of the total variance. In contrast to the full cranial and mandibular dataset, all marsupial taxa were concentrated in a small region of morphospace, near the origin, while the extremes of all axes, except for the positive end of PCO4, were defined by placentals.

PCO analysis of the early ossifying jaw bones for the subsampled dataset, with four ranks for each species, is again concentrated in the first few axes, with PCOs 1–4 representing 42.3%, 16.7%, 10.4%, and 8.1% of the total variance, respectively. As in the larger sample, marsupials were concentrated in a small region of morphospace (Fig. 3B), with all major axes defined at their extremes by placentals, except for the negative end of PCO4.

### Phenotypic trajectory analysis

Differences in method of data collection do not appear to have affected reconstructions of ontogenetic trajectories, with data from micro-CT scans, Reflex microscopy, and 3-D digitization all overlapping greatly (MANOVA *P* = 0.95). When analyzed with PCO1 and grouped by species, significant differences were observed based on species (*P* ≪ 0.01), ontogenetic rank (*P* = 0.008), and their interaction (*P* ≪ 0.001). There were no significant differences between pairs of species in ontogenetic trajectory direction or shape, but there were significant differences in trajectory size for comparisons involving, *Llama* and *Macropus*, which had significantly smaller trajectories than all other species other than each other and *Monodelphis* (Table 1). When taxa were grouped by infraclass, there were significant differences based on infraclass (*P* ≪ 0.01), but not on rank alone, nor on their interaction, and, for this reason, the results of this analysis are not discussed further.

When PCO1 is removed prior to PTA, there are significant differences based on species (*P* = 0.02) and on the interaction of species and rank (*P* ≪ 0.01), but not on rank alone (*P* = 0.12). When grouped by infraclass, there are significant differences based on infraclass (*P* = 0.03), but not on rank or their interaction.

When analyses are limited to the first ossifying bones of the oral apparatus (premaxilla, maxilla, and dentary), differences are significant based on species, rank, and their interaction (all *P* ≪ 0.01). Pairwise comparisons of species show many significant differences in trajectory shape and size (Supplementary Table S2). Of the 10 significant pairwise differences in trajectory shape, 5 differentiate placental taxa from marsupials, 4 differentiate between placentals, and only 1 differentiates between marsupials (*Phascolarctos* and *Macropus*). In trajectory size, all 12 significant differences distinguish marsupials from placentals, in all cases reflecting smaller trajectory size in marsupials than in placentals. Indeed, *Macropus, Trichosurus*, and *Phascolarctos* have shorter trajectories than all of the sampled placentals, and *Monodelphis* has a smaller trajectory size than all placentals except *Llama* (Table 1). *Phascolarctos* and *Llama* are also significantly different in trajectory direction. Of the 23 significant pairwise comparisons, then, 22 involve placentals. When grouped by infraclass, there are significant differences based on infraclass (*P* = 0.002), but not on rank or their interaction.

When the premaxilla, maxilla, and dentary data are analyzed without PCO1, there are again significant differences for species (*P* ≪ 0.01), rank (*P* = 0.013), and their interaction (*P* ≪ 0.01). There were no significant pairwise differences in trajectory direction, but many again for trajectory shape and size. Of the 10 significant differences in trajectory shape, 6 differentiate placentals and marsupials, 3 differentiate between placental species, and 1 differentiates between marsupial species. Eleven significant differences in trajectory size all discriminate between placental and marsupial taxa, and, in this case, all four marsupials have the shortest trajectories (Table 1). Thus, of 21 significant pairwise differences in oral bone trajectory shape and size, only one does not involve a placental taxon, and marsupials consistently show the smallest change in these bones through ontogeny, as reflected in trajectory size. As before, when grouped by infraclass, there are significant differences based on infraclass (*P* = 0.003), but not on rank or their interaction.

## Discussion

The results of these analyses support the hypothesis that marsupial taxa show less variation in ontogenetic trajectories for the early ossifying bones of the oral apparatus, but not for the cranium in general. There were few observed differences between marsupials and placentals in trajectory analyses of the full cranial dataset, and PCO analysis showed a great deal of overlap and similar morphospace occupation of marsupials and placentals on all major axes. In contrast, there are many significant differences in ontogenetic trajectory shape and size for the bones of the oral apparatus, and all but one of these involve placentals, either differentiating them from each other or from marsupials. These results suggest that, while marsupials are generally similar to each other in ontogenetic trajectory shape, size, and direction for the oral region, placentals are significantly more variable in trajectory shape and size.

Although the range of size change varies across the sampled taxa, due to the limitations of obtaining ontogenetic sequences from non-model organisms, these differences are unlikely to significantly alter these results. Most species are represented by a 2–4 times increase in skull length or body length (*Cavia, Dasypus, Llama*, *Rousettus*, *Peromyscus*, and *Macropus*), while sampled specimens of *Trichosurus* and *Talpa* specimens range in skull length by 1.52× and 1.37×, respectively, and *Monodelphis* and *Phascolarctos* specimens span >8× increase in skull length. Insofar as size increases reflect how much of ontogeny is sampled for each dataset, this variance in size ranges of specimens may impact comparisons of trajectory size. However, it is notable that none of the significant differences in trajectory size for the full dataset involve the taxa with particularly little or great size change sampled. For analyses limited to the early ossifying oral bones, the significant differences in trajectory size, all of which distinguish placentals and marsupials, may be due in part to differences in sampling, as 5 of the 12 significant differences involve *Trichosurus*. Nonetheless, significant differences between marsupials and placentals are overwhelmingly in the direction of longer trajectories in placentals, even when involving well-sampled marsupial taxa, such as *Monodelphis* and *Phascolarctos* (Table 1). Furthermore, significant differences in shape trajectories were not concentrated in taxa with smaller size ranges. For these reasons, we are confident that our results are robust to sampling and accurately reflect biological differences in the early craniogenesis of therian mammals.

The results of these analyses correspond well with previous analyses demonstrating that adult marsupials are less disparate in the morphology of the oral bones, but not that of the neurocranium, which ossifies after birth and is not expected to be under the same functional pressures as the bones involved in suckling (Bennett and Goswami 2013). Although it is not possible at present to estimate integration of these structures in most non-model organisms, due to the need for multiple specimens for each stage, these results also correspond well with the predictions of the previous analysis of integration in *Monodelphis*, which showed much higher integration of the oral bones in earliest stage of ontogeny sampled than was observed later in ontogeny in the oral bones, in any other cranial region for *Monodelphis*, or in any cranial region of the sampled placental, *Cryptotis*. Several studies have suggested that integration is repatterned through ontogeny (Zelditch 1988; Zelditch and Carmichael 1989b; Goswami et al. 2012) and that integration may decrease, while modularity increases, as individuals approach maturity (Zelditch and Carmichael 1989a; Goswami and Polly 2010b).

As Zelditch (1988) suggested, if strong integration among traits shapes their response to selection and limits variation to preferred directions of shape change, then changes in integration through ontogenetic time can have drastically different effects on ontogenetic trajectories and, more broadly, on morphological evolution. The marsupial reproductive strategy places their neonates under functional pressures that are dramatically different from those experienced by placental mammals. Having to propel into the pouch only a few weeks after conception, before most of the musculoskeletal system has even begun to develop hard tissues, and then suckling continuously for a period many times longer than gestation, places a great burden on the structures of the forelimb and the oral apparatus. The strong integration of the oral apparatus in early ontogeny may have evolved to ensure proper functioning during this important stage of development (Maier 1999), but it may also compound the functional constraints imposed on the marsupial oral apparatus, by redirecting any shape changes along limited trajectories compatible with the pattern of integration that dominates at that point in ontogeny. Both functional pressures and high integration independently may significantly limit variation, and their combination in the oral apparatus of marsupials early in ontogeny may constrain the evolution of that region more than if either factor were present in isolation.

It is important to note that marsupials, although today restricted in numbers and form relative to placentals, have outnumbered placentals in many regions in the past (Cifelli and Davis 2003). Fossil metatherians (marsupials and their stem relatives) display a greater diversity of cranial morphology and ecologies than observed in extant taxa (Wroe et al. 2005; Wroe and Milne 2007; Goswami et al. 2011; Bennett and Goswami 2013), although the oral apparatus of living and fossil marsupials combined is still significantly less disparate than that of placentals (Bennett and Goswami 2013). The results presented here contribute to a growing pool of evidence that the evolution of the marsupial oral apparatus has been constrained by their specialized mode of development. However, other factors, such as selective extinction and competition, have certainly contributed to their exclusion or near exclusion from certain regions (e.g., North America) where metatherians (marsupials and their stem relatives) previously dominated over contemporary eutherians (placentals and their stem relatives) (Cifelli and Davis 2003; Sánchez-Villagra 2013; Williamson et al. 2014). Nonetheless, these extrinsic factors do not explain the differential patterns for developmental and evolutionary variation observed in marsupial oral apparatus and the rest of the cranium. Thus, developmental constraints and the potentially aggravating factor of high integration during early ontogeny almost certainly have significantly limited the morphological evolution of marsupial oral apparatus, relative to other parts of their cranium and relative to that of their successful sister group, placentals.

### Future directions

As with many questions in biology, the relative importance of developmental constraints (i.e., functional pressures early in ontogeny) and of high integration in shaping morphological development and evolution can be difficult to discriminate. Each attribute independently may significantly limit variation on micro- and macroevolutionary scales, and both are present in the oral apparatus of marsupials early in ontogeny. Their interaction and relative contributions to patterns of morphological evolution may be addressed by looking at a taxon that has one but not both of these features (e.g., strong functional pressures at birth but low integration of the relevant bones, or vice versa). Peramelids are marsupials that do not undergo a lengthy crawl to the pouch (Lillegraven et al. 1987), and they have also been shown to deviate from other marsupials in forelimb ontogenetic trajectory (Sears 2004). Peramelids also wean earlier than other marsupials, and display faster cranial growth rates during the period of lactation, although their level of development at birth is similar to that of other marsupials (Gemmell et al. 1988). This shift in postnatal developmental rate does not appear to have resulted in any differences in postweaning growth in peramelids relative to other marsupials (Flores et al. 2013), and peramelids do suckle intensively during the shorter period of lactation, and so it is unlikely that they deviate from other marsupials in pattern of early cranial ontogeny. Previous study also suggests that they share the same general pattern of cranial integration as other marsupials (Goswami 2006, 2007). Nonetheless, this question is worth investigating further and with a more extensive dataset for both typical and atypical marsupials and placentals to better characterize the role of ontogenetic dynamics in shaping the morphological diversification of mammals. In particular, it would be ideal to have large sample sizes for individual ontogenetic stages for a diversity of taxa, rather than the few model organisms that are available at present, to establish that marsupials and placentals systematically differ in patterns of ontogenetic integration. More broadly, other tetrapod taxa would also be worthwhile to investigate, as many other clades experience strong functional pressures early in ontogeny but may not show similar patterns of ontogenetic integration to the examined mammals.

Even without better ontogenetic samples, simulations and quantitative analyses may be useful in further clarifying the relationships among ontogenetic integration, developmental constraints, and large-scale patterns of morphological evolution. For example, as discussed above, simulations have already been used to define expectations of morphological change for empirically-derived covariance matrices (Hansen and Houle 2008; Marroig et al. 2009; Shirai and Marroig 2010; Goswami et al. 2014; Linde-Medina et al. forthcoming 2016). One could use this approach to assess whether the observed covariance structures at different ontogenetic stages and for different cranial regions align with actual variation in cranial morphology in marsupials. Additionally, one could model the effects of selection at different points in ontogeny, using the relevant covariance structures at those stages, to generate expectations for the impact of changing ontogenetic integration on morphological variation. The hypotheses and empirical analyses presented here represent an important step toward bridging these topics with ontogenetic data for two relatively diverse sister clades, but future work with both empirical and theoretical approaches will be crucial to further defining these effects and their broader significance.

## Supplementary Material

Supplementary Data

## Supplementary Data

Supplementary Data available at *ICB* online.
