# Supplementary material for: Do Developmental Constraints and High Integration Limit the Evolution of the Marsupial Oral Apparatus?
Source: Integr Comp Biol. 2016 Jun 2;56(3):404–15. doi: 10.1093/icb/icw039 (PMC4990707; doi:10.1093/icb/icw039)
Supplement: Supplementary Data [file supp_56_3_404__index.html]

Do Developmental Constraints and High Integration Limit the Evolution of the Marsupial Oral Apparatus? — Do Developmental Constraints and High Integration Limit the Evolution of the Marsupial Oral Apparatus? — Do Developmental Constraints and High Integration Limit the Evolution of the Marsupial Oral Apparatus? — Supplementary Data 

# Do Developmental Constraints and High Integration Limit the Evolution of the Marsupial Oral Apparatus?

## Supplementary Data

files

- Supplementary Data - xlsx file
